# GlyComb: A novel glycoconjugate data repository that bridges glycomics and proteomics

**DOI:** 10.1016/j.jbc.2023.105624

**Published:** 2024-01-03

**Authors:** Yushi Takahashi, Masaaki Shiota, Akihiro Fujita, Issaku Yamada, Kiyoko F. Aoki-Kinoshita

**Affiliations:** 1 Department of Bioinformatics, Graduate School of Engineering, Soka University, Tokyo, Japan; 2 Glycan and Life Systems Integration Center, Faculty of Science and Engineering, Soka University, Tokyo, Japan; 3 Laboratory of Glycoinformatics, The Noguchi Institute, Tokyo, Japan

**Keywords:** bioinformatics, glycobiology, glycoconjugate, glycomics, glycoprotein, proteomics, repository

## Abstract

The glycosylation of proteins and lipids is known to be closely related to the mechanisms of various diseases such as influenza, cancer, and muscular dystrophy. Therefore, it has become clear that the analysis of post-translational modifications of proteins, including glycosylation, is important to accurately understand the functions of each protein molecule and the interactions among them. In order to conduct large-scale analyses more efficiently, it is essential to promote the accumulation, sharing, and reuse of experimental and analytical data in accordance with the FAIR (Findability, Accessibility, Interoperability, and Re-usability) data principles. However, a FAIR data repository for storing and sharing glycoconjugate information, including glycopeptides and glycoproteins, in a standardized format did not exist. Therefore, we have developed GlyComb (https://glycomb.glycosmos.org) as a new standardized data repository for glycoconjugate data. Currently, GlyComb can assign a unique identifier to a set of glycosylation information associated with a specific peptide sequence or UniProt ID. By standardizing glycoconjugate data via GlyComb identifiers and coordinating with existing web resources such as GlyTouCan and GlycoPOST, a comprehensive system for data submission and data sharing among researchers can be established. Here we introduce how GlyComb is able to integrate the variety of glycoconjugate data already registered in existing data repositories to obtain a better understanding of the available glycopeptides and glycoproteins, and their glycosylation patterns. We also explain how this system can serve as a foundation for a better understanding of glycan function.

Glycosylation reactions are biochemical processes in which monosaccharides and oligosaccharides are joined to macromolecules including lipids and proteins. Transfer of glycans to proteins is a co- and post-translational modification of which there are a number of classes (1). There are several well-known classes of protein glycosylation: glycans attached to the nitrogen atoms of asparagine residues in amino acid sequences are called *N*-linked glycans, while glycans attached to the oxygen atoms of serine or threonine residues are called *O*-linked glycans (2). It is known that glycosylated proteins are presented on the cell surface or secreted out of the cell to mediate various types of intercellular communication, cell-extracellular matrix interactions, and cell-molecule interactions (3). Thereby, glycosylation to proteins and lipids is reported to be closely related to the mechanisms of various diseases such as influenza, cancer, and muscular dystrophy (4, 5, 6). Therefore, it is now recognized that the analysis of post-translational protein modifications, including glycosylation, is essential to accurately understand the functions of each protein molecule and the interactions among them. Recent advances in glycoproteomics have led to the mapping of *N*-glycosylation sites in biofluids and cellular materials obtained from patients, implying that the occupancy of *N*-glycosylation sites in proteins and the signatures of glycopeptides can be used as biomarkers for the diagnosis of neurological diseases such as Alzheimer's disease and for the early detection of cancer (7, 8, 9).

In order to efficiently perform large-scale analyses to determine how the presence or absence of glycosylation of specific sites of a particular protein causes differences in its function, it is essential to promote the accumulation, sharing, and reuse of experimental and analytical data in accordance with the FAIR (Findability, Accessibility, Interoperability, and Re-usability) data principles (10). With this purpose, the development and use of public data repositories are recommended in the life science field (11). In the field of glycoscience, several public data repositories have been developed including the international glycan structure repository GlyTouCan (12), GlycoPOST (13) for depositing raw data obtained from mass spectrometry experiments in glycomics, and UniCarb-DR (14) for depositing annotated glycomics data, which allows visualization using the GlycoWorkbench software (15). In particular, GlyTouCan assigns a unique identifier to each glycan structure based on WURCS (16), which is a glycan structure notation that allows a unique linear representation of a single glycan structure, which could be a monosaccharide composition. The glycan structure assigned an identifier by GlyTouCan is published on the Internet as Linked Open Data (17) so that researchers can easily retrieve other omics information other than glycomics related to the glycan structure by performing a search utilizing Semantic Web technologies. GlyTouCan has been adopted as the recommended repository for glycan data and is used to integrate glycan data in the GlySpace Alliance (18), which includes GlyCosmos (19), GlyGen (20), and Glycomics@ExPASy (21). On the other hand, GlycoPOST accepts and accumulates raw data generated by glycomics mass spectrometry experiments from researchers around the world according to the MIRAGE (Minimum Information Required for a Glycomics Experiment) guidelines (22, 23, 24).

However, glycans do not function alone; they serve as modulators of protein functions. Thus glycoproteomics is an important field for understanding how proteins are modulated by glycans. With current technologies, glycoproteins are identified using various mass spectrometry techniques combined with orthogonal methods, but in the end, the data usually consist of identified peptides and glycosylation sites annotated with one or more monosaccharide compositions (hereafter referred to as glycan structures). In order to integrate this data with current protein-centric and glycan-centric databases, it is crucial to have a standardized system to identify glycoconjugates, and in particular glycoproteins and glycopeptides. Such a public data repository for storing and sharing glycoconjugate data, including glycopeptides and glycoproteins, was lacking, thus hindering the efficient accumulation of glycoconjugate knowledge. It is true that the UniProtKB database (25), for example, contains glycosylation information for each protein entry. However, because UniProtKB assigns an identifier only at the protein level, while each glycosylation site may have a publication reference, it is currently not possible to search for a specific set of glycosylation patterns on a particular peptide sequence, which may have particular relevance to a disease state. Glycopeptide and glycoprotein profiles identified in glycoproteomics experiments reported in the literature are often compiled into MS Excel or CSV files and either provided as Supplementary Data or uploaded together with raw data of liquid chromatography-tandem mass spectrometry (LC-MS/MS) experiments to a proteomics data repository participating in the ProteomeXchange Consortium (26) such as Proteomics Identification Database (PRIDE) (27). However, since there is no standardized format for these files, each researcher submits their own data in their preferred format. This means that in order to collect and analyze experimental results containing specific glycopeptides or glycoproteins from such data repositories, these uniquely formatted files must be opened and processed individually, which is a time-consuming process.

To overcome these issues, we have developed GlyComb as a repository for depositing glycoconjugate data to accelerate glycoscience research. The data deposited in GlyComb will enable the unique identification of glycopeptides and glycoproteins contained in glycoproteomics experiment results submitted to GlycoPOST, and to link the glycan data with GlyTouCan identifiers. Currently, GlyComb can assign a unique identifier to a set of glycosylation information associated with a specific peptide sequence or UniProt ID, which can be registered and published as a glycopeptide or glycoprotein entry, respectively. GlyComb is now publicly available on the Internet (https://glycomb.glycosmos.org), and users can access this new web resource using a web browser. We have aimed to make GlyComb user-friendly, providing tools that make it easy to upload glycopeptide and glycoprotein information to register.

Here we first describe the data format for submission to GlyComb and the overall workflow for data submission and data publishing. We also introduce a data conversion utility to automatically extract glycoconjugate entries for submission to GlyComb from the summary spreadsheet generated by PMI-Byonic (28), which is a software often used for mass spectrometry experimental analysis in glycoproteomics, and present some statistics of the data currently registered in GlyComb. Finally, we will discuss some use cases for GlyComb, followed by plans and expectations for its future usage.

## Results

### Input data format for GlyComb

**Figure 1:**
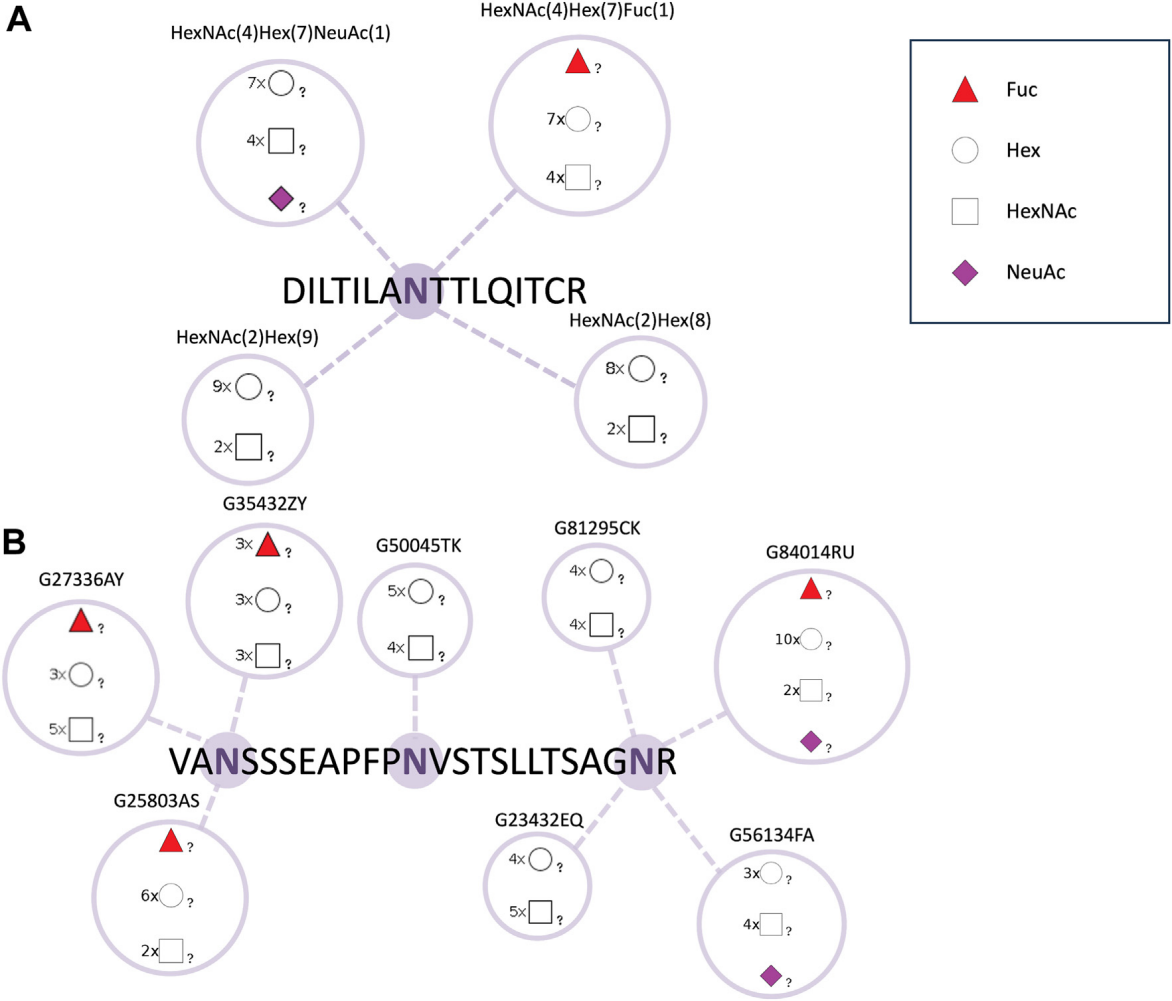
**Conceptual diagram of glycopeptides illustrated using SNFG monosaccharide notation symbols**. The "?" character assigned to each symbol indicates the linkage information (number of backbone carbon atoms participating in the covalent bond and anomeric configuration) of the monosaccharide molecule is unknown. Glycoproteomics experiments may result in multiple potential glycosylation possibilities for the same amino acid residue on a peptide obtained by protease treatment of the protein sequence with trypsin or other proteases. For each glycan structure represented symbolically, only the monosaccharide composition information is provided instead of the exact molecular structure using (*A*) PMI Byonic-like notation and (*B*) GlyTouCan IDs. In GlyTouCan, each glycan structure assigned a GlyTouCan ID is described using WURCS format, which can also express glycan composition information. Therefore, GlyTouCan IDs can also be used to represent glycan composition information.

LC-MS/MS is now the primary method used by researchers around the world for glycoproteomics experiments to determine which residues in peptide and protein amino acid sequences are modified with which glycan structures (29). Because monosaccharides generally have multiple isomers (*e.g.*, D-glucose, D-galactose, D-mannose, etc.), it is very difficult to assign a specific isomer to each monosaccharide in a glycan for the molecular weight observed in an experiment. Therefore, most of the results obtained from this mass spectrometry-based approach only contain the monosaccharide composition that constitutes a glycan molecule, and not the fully-defined structure of the glycan molecule (30). Figure 1*A* shows a conceptual diagram of a glycopeptide using the Symbol Nomenclature for Glycans (SNFG) (31) monosaccharide symbol notation.

In glycomics, various glycan structure notations have been developed, including the IUPAC-condensed format, GlycoMinds LinearCode format (32), KEGG Chemical Function (KCF) format (33), GlycoCT format (34), and WURCS format. Among these notations, in particular, the WURCS format has been adopted by GlyTouCan because it can uniquely represent any glycan structure in a linear format. In addition to glycan structures for which all monosaccharide and linkage information are identified, WURCS format can also uniquely represent glycan structures for which linkage information is unknown or for which only monosaccharide composition information is known, such as those obtained from mass spectrometry experiments. GlyTouCan assigns an individual GlyTouCan ID to each glycan structure that can be represented uniquely in WURCS format. Figure 1*B* shows a conceptual diagram of a glycopeptide in which the glycan structures modified on the peptide are represented by GlyTouCan IDs.

**Figure 2:**
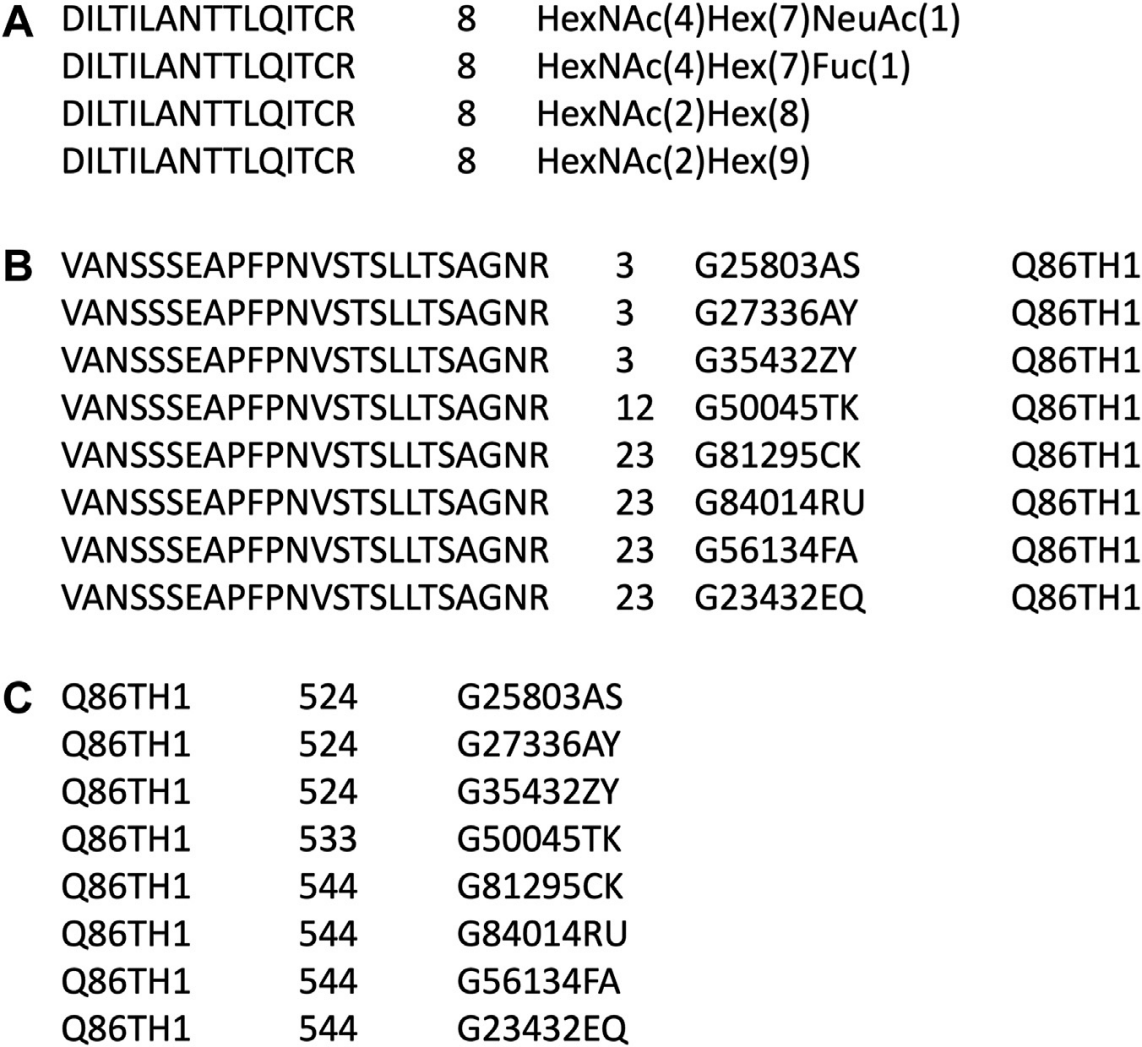
**Examples of glycopeptide and glycoprotein information that can be input into GlyComb.***A*, example of glycopeptide input entry to GlyComb using PMI Byonic-like composition notation of glycan structures, corresponding to the conceptual diagram of the glycopeptide shown in Figure 1*A*. *B*, example of a glycopeptide input entry to GlyComb representing a glycopeptide described using the GlyTouCan IDs shown in Figure 1*B*. In the glycopeptide input, a UniProt ID can also be optionally specified in the fourth column. Detailed information on the glycan structures corresponding to each GlyTouCan ID is available at https://glytoucan.org. *C*, example of a glycoprotein entry to enter into GlyComb described using GlyTouCan IDs.

In GlyComb, we adopted a tab-separated values (TSV) format in which the amino acid sequence of a peptide, the residue numbers of glycan modifications, and the glycan structures are delimited by tab characters as the input format for submitting glycopeptide entries. For glycoprotein entry submissions, we adopted a similar TSV format, in which the UniProt ID for the protein, the modified residue numbers, and the glycan structures are delimited by tab characters. If a modified residue number is unknown, users can use the "?" character instead of the residue number. For the glycan representation, we adopted a glycan composition notation similar to that used in PMI-Byonic as one of the glycan structure notations. Moreover, to be able to accept ambiguous linkage information and accurate monosaccharide composition information, GlyTouCan IDs can be used to specify the glycan structures. Thus currently, GlyComb accepts a peptide sequence or a UniProt ID and a set of related glycosylation information - that is, the number of the glycosylated residue and the glycan structure modifying it - as a valid GlyComb entry and assigns a unique GlyComb ID to it. Figure 2, *A* and *B* show examples of valid glycopeptide inputs to GlyComb, described using two different glycan structure notations. As shown in Figure 2*B*, additional UniProt ID information can be specified in the fourth column for glycopeptide entries to specify the source protein of the specified peptide sequence. Figure 2*C* shows an example of a glycoprotein entry in GlyComb. Unlike glycopeptide entries, glycoprotein entries contain a UniProt ID in the first column. Since protein sequence information corresponding to a UniProt ID is occasionally updated, changed, inactivated, or deleted in UniProt, glycoprotein information submitted with a UniProt ID is subject to strict validation by referring to the exact sequence information during the registration process in GlyComb.

### Workflow for submitting and publishing data to GlyComb

GlyComb is currently available on the Internet, and researchers can submit their glycopeptide and glycoprotein entries in the format described in the previous section at https://glycomb.glycosmos.org/registration after user login with their own Google account. If they are not logged in, they can only browse the list of entries that have already been published. In addition, in order to accept input from researchers who do not have a Google account, we plan to add login methods other than Google accounts, such as identifiers issued by the Open Researcher and Contributor ID (ORCID) (35).

**Figure 3:**
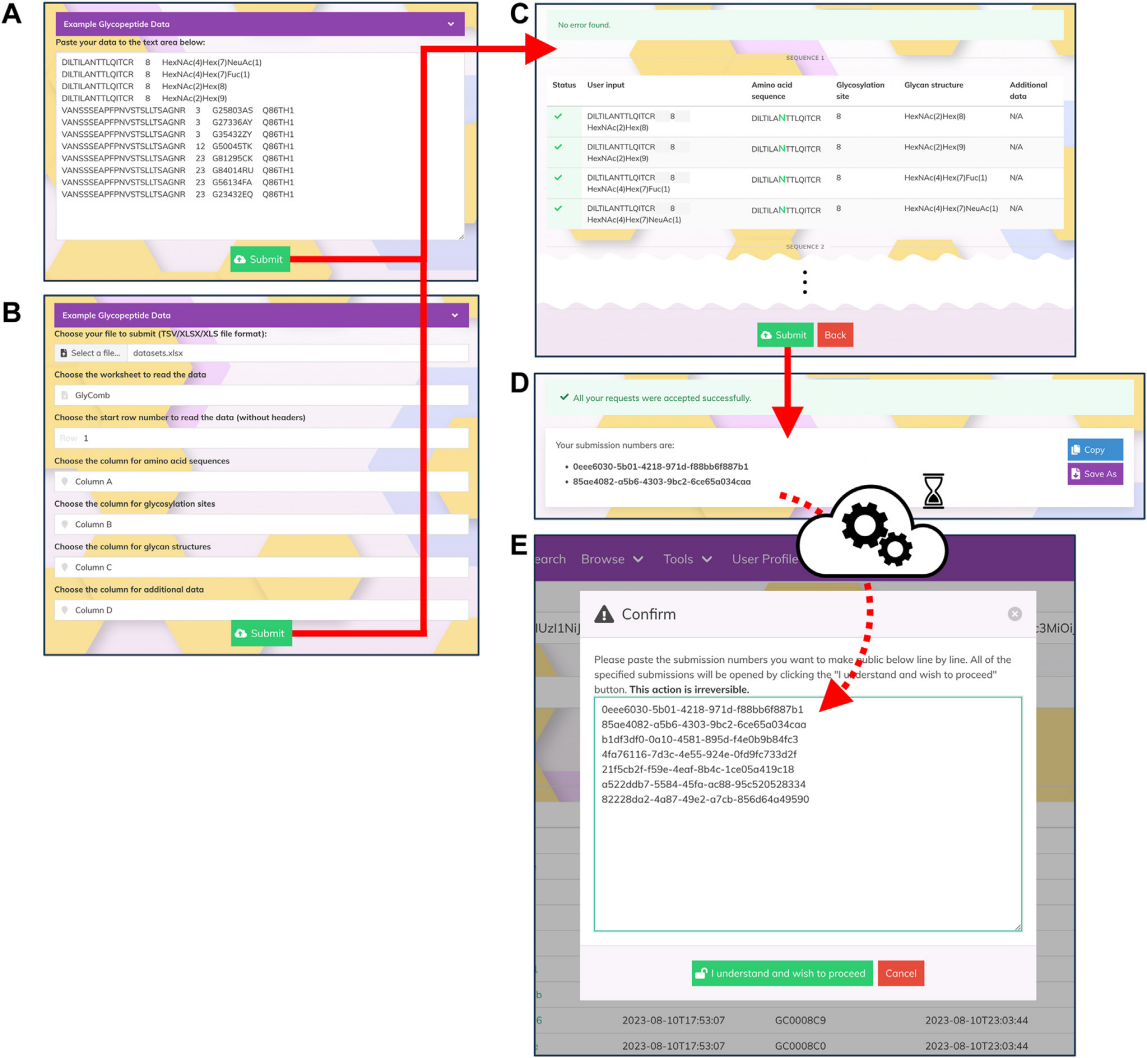
**Workflow for registering and making glycopeptide entries publicly available in GlyComb**. First, researchers access the entry registration screen (https://glycomb.glycosmos.org/registration) after logging into GlyComb with their Google account. They can either (*A*) copy and paste the entries they want to register from the clipboard into the text area on the screen or (*B*) select an MS Excel file or TSV file to upload. When uploading a file, they can choose the worksheet to be read or select the columns to be read. *C*, a confirmation screen to verify that the submission content is correct will be displayed. *D*, GlyComb displays a submission number instead of the GlyComb ID, which is the accession number, when the submission of an input entry is completed. By using these submission numbers, researchers can later make their registered entries open to the public. *E*, a confirmation screen to make each registered entry available to the public (https://glycomb.glycosmos.org/user_profile). Through batch processing on the GlyComb server, they are assigned unique GlyComb IDs within a few hours after the input entries are submitted and each entry is ready to be published. By entering the submission numbers generated when the glycopeptide or glycoprotein entries were submitted, one per line, and clicking the button at the *bottom* of the pop-up window, researchers can make multiple GlyComb entries they have submitted open to the public at once.

GlyComb provides two ways for researchers to submit their input entries: copy and paste input from the clipboard using the text area on the screen (Fig. 3*A*) or by file upload of TSV or MS Excel files (Fig. 3*B*). Multiple entries can be submitted at once by consecutively specifying them in the same file or text area. Blank lines can be included between the entries. Since GlyComb assigns different GlyComb IDs to different peptide sequences or UniProt IDs, GlyComb automatically distinguishes different peptide sequences and UniProt ID when multiple input entries are submitted at once. As shown in Figure 3*C*, a confirmation screen is displayed when submitting input data, allowing researchers to confirm their submission to GlyComb after their submission.

When input entries are submitted to GlyComb, researchers will first receive submission numbers for each input entry (Fig. 3*D*). These submission numbers can be downloaded and saved as a text file. Instead of immediately issuing a GlyComb ID for each registered entry, GlyComb adopts a batch validation system whereby input entries are validated together later during batch processing before accession numbers are issued. Furthermore, glycopeptide and glycoprotein entries assigned GlyComb IDs are not automatically opened to the public, and each GlyComb entry with a GlyComb ID will not be opened to the public unless the researcher who registered the entry explicitly follows the publication procedure for each entry using the submission number issued when the registration was completed. Each entry can be made public from the user profile screen, which can be accessed via the URL https://glycomb.glycosmos.org/user_profile after logging in to GlyComb. Figure 3*E* shows the form for making each entry public. By clicking on the "Open to the public" button on the screen, an input form for entering the submission number corresponding to the entry the user wishes to make public will be displayed. By entering the submission numbers on each line in this text area and clicking on the "I understand and wish to proceed" button, the GlyComb entry publication procedure will be completed. If other researchers have already registered the exact same glycopeptide or glycoprotein entries in GlyComb, each entry will be assigned the same GlyComb ID. It is of course possible for a researcher to publish his/her own entry, which is one of those entries, even if other researchers have not published them. GlyComb guarantees that entries assigned a GlyComb ID will not have their content changed or deleted at all in the future. This means that even if researchers notice a mistake in an entry once they have registered it, they will not be able to update its content. Therefore, if there is an entry in GlyComb that contains any errors, it must be re-registered with the corrected data as a new entry in GlyComb. In fact, we assume that registering data in GlyComb is only the initial phase of the accumulation of glycoconjugate information. As a next phase, we plan to curate and annotate the deposited data and link it to existing knowledge in knowledge bases such as GlyCosmos. There, it is expected that only accurate data, or data with evidence, will be incorporated. Consequently, incorrect data will be left as unreferenced information in the GlyComb repository.

### Automatic extraction of glycopeptide and glycoprotein entries from summary worksheets generated by PMI-Byonic

In order to assist users in uploading their glycoproteomics data into GlyComb, we decided to provide utility software to convert glycopeptide and glycoprotein identification results generated from glycoproteomics experimental analysis software tools commonly used by glycoproteomics researchers directly into a set of input entries for GlyComb. As a first step, we have implemented and embedded into the GlyComb system a conversion software to extract input entries for GlyComb from the summary spreadsheet file generated from PMI-Byonic. This software is now available at https://glycomb.glycosmos.org/byonic-summary-worksheet-converter and can be used in web browsers.

**Figure 4:**
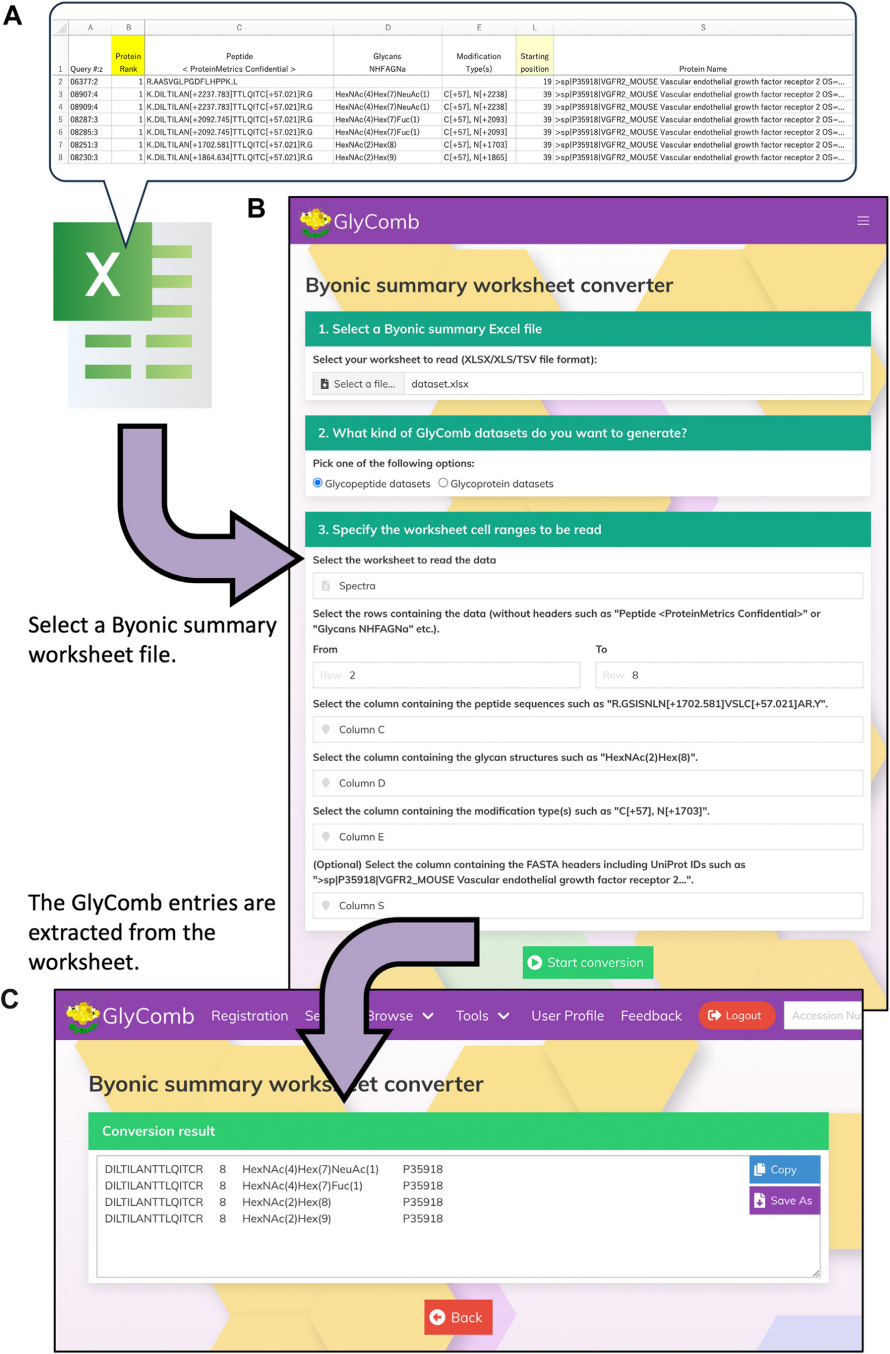
**The overall workflow for extracting input entries for GlyComb from a PMI-Byonic summary spreadsheet.***A*, example summary worksheet of a glycoproteomics experiment generated from PMI-Byonic. This data was prepared by partially modifying the contents of a Byonic summary spreadsheet contained in one of the datasets published in the PRIDE Data Repository as PXD012629 (https://www.ebi.ac.uk/pride/archive/projects/PXD012629). Users can first select which entry to generate, glycopeptide entry or glycoprotein entry, in our conversion software. In order to generate glycopeptide entries for GlyComb, the peptide sequence information contained in column C, the modified glycan structure information contained in column D, and the type of modification information contained in column E in this worksheet are required. On the other hand, to generate a glycoprotein entry, the peptide start residue number in the amino acid sequence of the protein in column L and the protein name containing a UniProt ID in column S are additionally required. In order to determine the residue to which the glycan structure is modified, this software compares the mass information of the modification and looks for a residue whose mass information of the modification to the amino acid residue is very close (this threshold value is set to <1.0). Taking into account that some Byonic summary spreadsheets provide the mass of the glycan structure as an integer, we have determined the value of this threshold. *B*, by selecting one spreadsheet file, a form for specifying whether to generate glycopeptide or glycoprotein entries and setting the range of data to be read will be displayed. *C*, the conversion results of the selected spreadsheet information can be easily copied to the clipboard or saved as a text file. In this example, we specified that rows 2 through 8 in the selected worksheet are to be read, and any duplicate results of each row are automatically removed from the conversion results. In the same way, rows in the worksheet that do not have glycosylation information are not reflected in the conversion result.

PMI-Byonic performs *in silico* variable modification searches of protein sequences against reference databases to identify glycopeptide sequences, and outputs sequence information with sufficient peptide fragment ion evidence as a peptide-spectrum match (PSM) into the summary spreadsheet (28, 36). Figure 4 shows the workflow of our conversion software. First, as shown in Figure 4*A*, users will select an Excel file generated from PMI-Byonic that contains PSM information, including peptide sequences with modified residue positions, modified glycan structures and their masses, and type of modification information. If they utilize this software to generate glycoprotein entries, they will additionally need the residue number on the protein where the peptide sequence starts and the name of the protein containing a UniProt ID. When a worksheet is selected, a data range setting form is displayed, as shown in Figure 4*B*. With this form, users can choose whether to generate glycopeptide or glycoprotein entries, select the worksheets to be scanned in that spreadsheet file, and specify the columns that contain each kind of information. After filling out this form, by clicking the button at the bottom of the screen, the conversion will be executed and the conversion results will be displayed as shown in Figure 4*C*.

When extracting glycoprotein entries, this software automatically retrieves the amino acid sequence information corresponding to the UniProt IDs in the spreadsheet from the UniProt database. Then, the glycosylated peptide sequence information is matched with the residue number information where that peptide sequence starts on the protein to ensure that this information is consistent. This ensures that users will generate glycoprotein entries for proteins having valid UniProt IDs. As for the substituent information in glycan structures, this software currently supports only two substituents, "Phospho" and "Sulfate". This is due to the limited number of substituent types supported by the program provided by GlyCosmos for converting glycan composition strings to WURCS format, which is used by our software to verify whether the detected glycan composition strings are in a valid format. We plan to address other substituents such as "Methyl" and "Acetyl" in collaboration with the GlyCosmos development team. During the conversion process, communications with the GlyCosmos server occur to check whether the GlyTouCan IDs have already been assigned for each glycan structure. However, all other information in the spreadsheet selected by the user will not be sent to the server side. Therefore, all conversion processes are performed locally in the user's web browser.

Although PMI-Byonic is indeed a software used by a large number of glycoproteomics researchers, it is a paid software and not all researchers use it. In addition to commercial software such as PMI-Byonic and Mascot (37), other freely available software such as Protein Prospector (38) and GlycReSoft (39) are also being used to analyze the results of glycoproteomics experiments. Therefore, to encourage more researchers to submit glycoproteomics data to GlyComb, we plan to add more conversion software so that identification results generated from a wide range of glycoproteomics software can be input into GlyComb.

### Registration of glycopeptide and glycoprotein entries extracted from the PRIDE and MS-Viewer repositories

**Figure 5:**
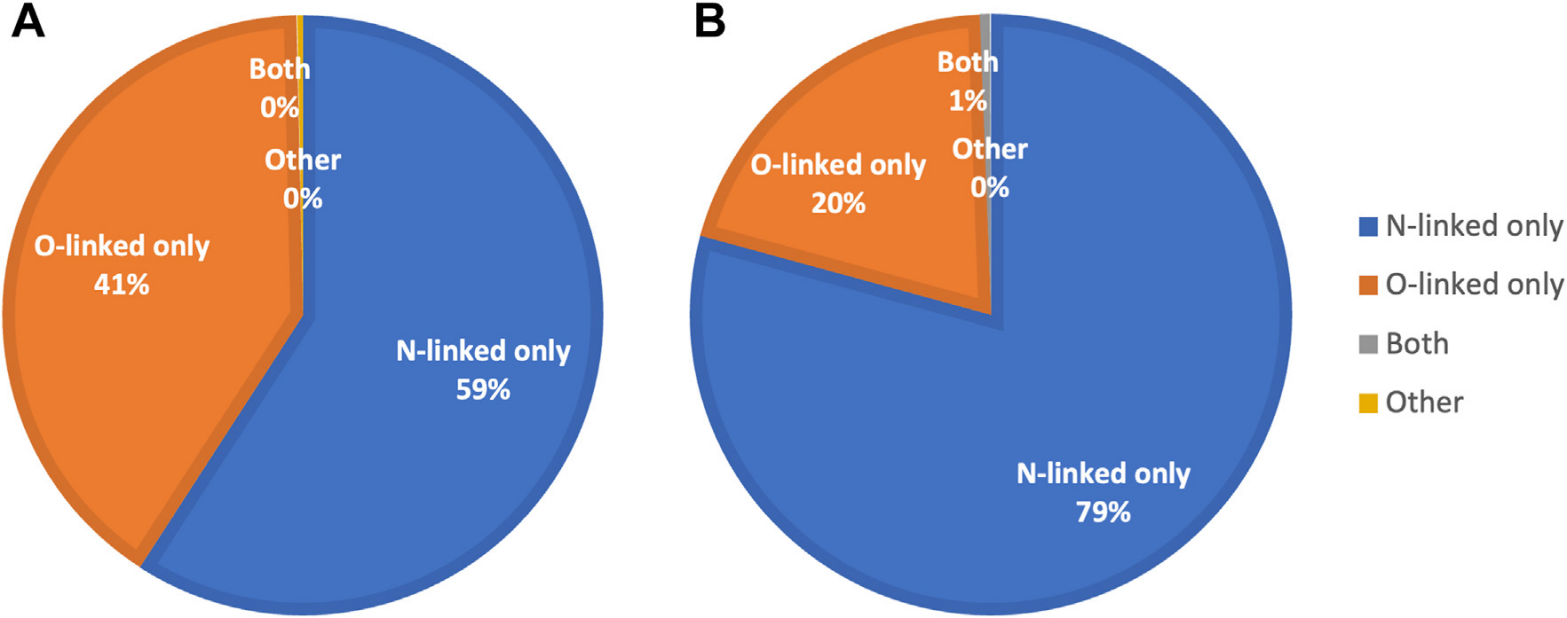
**Percentage of glycopeptide and glycoprotein entries registered in GlyComb categorized by glycosylation class as of August 31, 2023.** There are 99,240 glycopeptide entries registered in GlyComb in total, of which (*A*) 59% (58,682 entries) contain only N-linked glycosylation and 41% (40,203 entries) contain only O-linked glycosylation. There were 74 registered glycopeptide entries containing both *N*-linked and *O*-linked glycosylation, and 281 glycopeptide entries containing neither of these. For glycoprotein entries (*B*), a total of 26,953 glycoprotein entries were registered in GlyComb, of which 79% (21,359 entries) contained only *N*-linked glycosylation and 20% (5440 entries) contained only *O*-linked glycosylation. There are 137 registered glycoprotein entries that contain both *N*-linked and *O*-linked glycosylation, and 17 entries that are neither of these. There are 40 glycopeptide entries with UniProt IDs among the 281 glycopeptide entries having neither *N*-linked nor *O*-linked glycosylation, whereas the remaining 241 entries do not contain any UniProt ID information. Among these 40 entries, 30 entries are derived from 17 glycoprotein entries having neither *N*-linked nor *O*-linked glycosylation, while the remaining 10 entries form *N*-linked or *O*-linked glycoproteins with other glycopeptide entries. Furthermore, out of these 281 glycopeptide entries, the modified residues of 270 entries are undefined, while the modified residues of the remaining 11 entries are amino acid residues other than asparagine, serine, and threonine. Two of these entries are considered *C*-Man modified to tryptophan residues, while the remaining nine entries are modifications to other amino acid residues, including alanine, arginine, and tyrosine.

Figure 5 shows a breakdown of glycopeptide and glycoprotein entries registered in GlyComb as of August 31, 2023. In order to collect glycopeptide and glycoprotein information identified from existing glycoproteomics studies available on the Internet, we surveyed a number of data repositories for proteomics experiments that are members of the ProteomeXchange Consortium. We found that identification results were uploaded as comma-separated values (CSV), TSV, or MS Excel files in each contributor's own format. First, in order to extract glycopeptide and glycoprotein entries from those research results using the conversion software mentioned in the previous section, we collected PMI-Byonic-generated identification summary spreadsheets of glycoproteomics experiments from the submissions published in the PRIDE database. As a result, we obtained 1465 PMI-Byonic-generated summary spreadsheet files from 24,145 submissions currently available in the PRIDE Archive (https://www.ebi.ac.uk/pride/archive/) as of August 8, 2023. We generated input datasets for GlyComb from these spreadsheet files using our conversion software, resulting in 95,125 unique glycopeptide entries and 24,831 unique glycoprotein entries.

In addition, we examined 683 entries corresponding to MS-Viewer Keys published in the MS-Viewer (40) repository (https://msviewer.ucsf.edu/prospector/cgi-bin/msform.cgi?form=msviewrep), which is built into Protein Prospector, a suite of proteomics experimental analysis software and database. As a result, we successfully obtained a total of 3189 glycopeptide entries that can be entered into GlyComb and 1096 glycoprotein entries that can be entered into GlyComb out of 19 MS-Viewer entries. The glycosylation information published in the MS-Viewer repository included ambiguous data that had multiple candidates for the amino acid residue number to be modified in the peptide sequence, as well as multiple candidates for the glycan structure to be modified. This ambiguous glycosylation information was excluded from the conversion candidates when we extracted the input entries to GlyComb. We are planning to extend the GlyComb system so that such ambiguous data can also be stored in GlyComb in the future.

**Figure 6:**
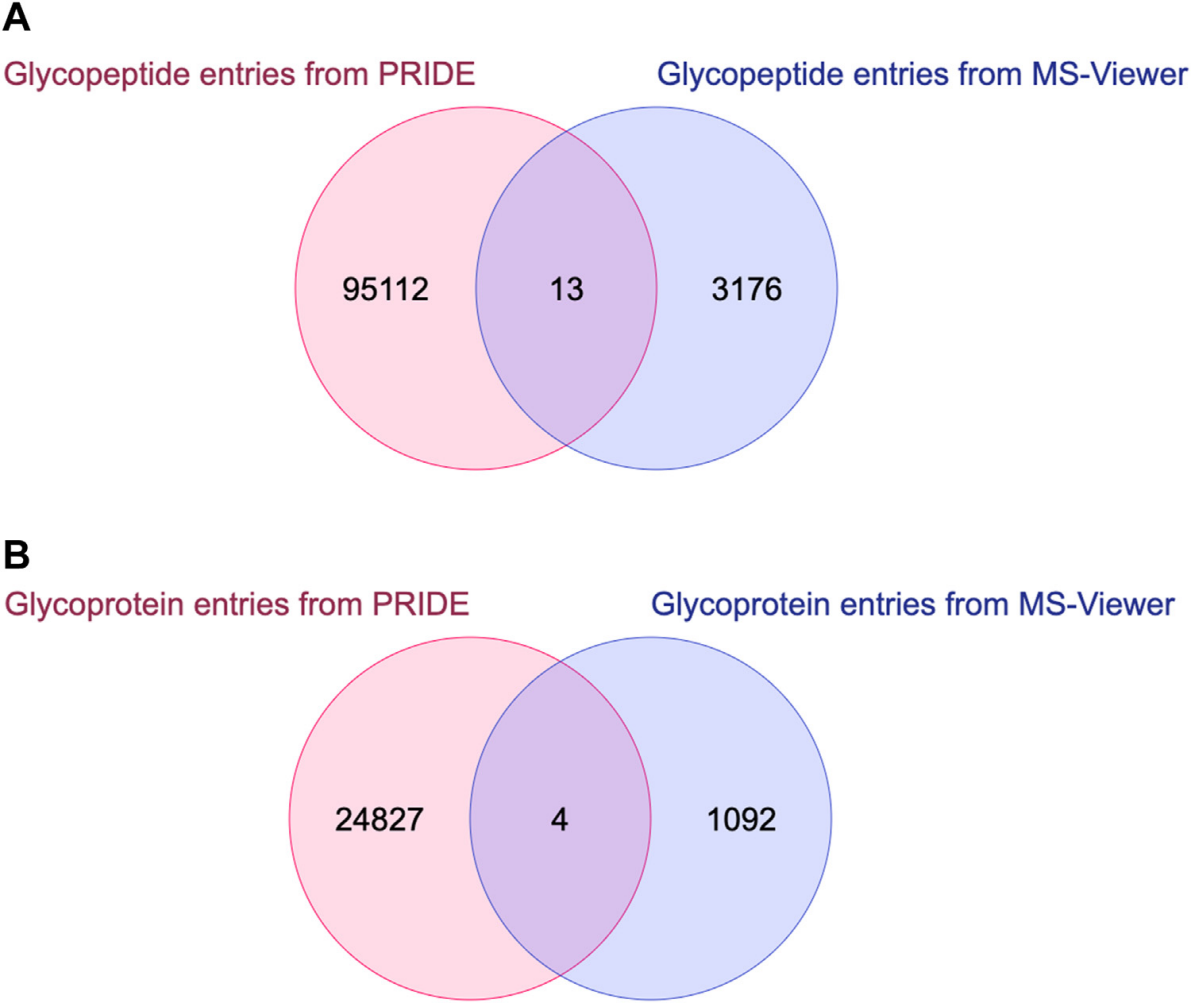
**Venn diagrams showing the number of overlapping glycopeptide and glycoprotein entries extracted from two different data repositories.***A*, a Venn diagram showing the number of glycopeptide entries extracted from PRIDE and MS-Viewer overlapped with each other, and (*B*) a Venn diagram showing the number of glycoprotein entries extracted from each repository overlapped with each other.

After registration of all of these data, each entry was assigned a GlyComb ID. Based on the assigned IDs, we investigated how many entries extracted from the PRIDE repository overlapped with those from the MS-Viewer repository (Fig. 6). As a result, we found that 13 glycopeptide entries and 4 glycoprotein entries overlapped. Such a comparison would have been extremely difficult to perform without these GlyComb IDs. Table 1 is a breakdown of these overlapped glycopeptide entries, showing the GlyComb ID of each entry, the corresponding accession numbers of the PRIDE and MS-Viewer repositories containing them, and the associated UniProt IDs.

**Table 1:** Breakdown of the 13 overlapping glycopeptide entries extracted from the PRIDE and MS-viewer repositories

GlyComb ID	Glycopeptide entry	PRIDE project accession ID	MS-Viewer accession ID (MS-Viewer key)	UniProt ID(s); protein name
GC001E29		PXD018048	PPV000097 (xjfsua8jyd)	P05154; Human plasma serine protease inhibitor
GC001E35		PXD018048	PPV000097 (xjfsua8jyd)	P02765; Human alpha-2-HS-glycoprotein
GC001E55		PXD018048	PPV000108 (ix1tmqvo4h)	P05154; Human plasma serine protease inhibitor
GC00A221		PXD020196	PPV000002 (dil8ljwmkv)	Q9Z1M0, A2VCP3; Mouse P2X purinoceptor 7, Mouse P2X purinoceptor
GC00A31 C		PXD020196	PPV000002 (dil8ljwmkv), PPV000046 (tbguwfn09u)	P97797; Mouse tyrosine-protein phosphatase non-receptor type substrate 1
GC00A8D9		PXD020196	PPV000045 (qunwxqxpy4), PPV000094 (ncoivkfko4)	P97426; Mouse eosinophil cationic protein 1
GC00B193		PXD020648	PPV000097 (xjfsua8jyd)	P02649; Human apolipoprotein E
GC00BCAE		PXD023943	PPV000097 (xjfsua8jyd)	P04196; Human histidine-rich glycoprotein
**GC017098**		PXD035445	PPV000046 (tbguwfn09u)	Q02819; Mouse nucleobindin-1
**GC0170AE**		PXD035445	PPV000002 (dil8ljwmkv), PPV000045 (qunwxqxpy4)	Q02819; Mouse nucleobindin-1
GC0170B3		PXD035445	PPV000002 (dil8ljwmkv)	P12023, Q6GR78; Mouse amyloid-beta precursor protein, Mouse amyloid-beta A4 protein
GC017134		PXD035445	PPV000046 (tbguwfn09u)	P24593, Q07079; Human insulin-like growth factor-binding protein 5, Mouse insulin-like growth factor-binding protein 5
GC0173AC		PXD035445	PPV000046 (tbguwfn09u)	P12023; Mouse amyloid-beta precursor protein

Note that there are two GlyComb ID pairs, GC017098-GC0170AE (bold) and GC0170B3-GC0173AC (underlined), sharing the same peptide sequence, but they were assigned different GlyComb IDs because of the different glycosylation patterns. By assigning IDs to these glycopeptide entries, GlyComb could facilitate comparison of the results of glycoproteomics experiments submitted to different data repositories.

## Discussion

With the development of GlyComb, the first data repository for glycoconjugate data including glycopeptide and glycoprotein data from glycoproteomics studies, we believe that glycobiology research can be accelerated by combining knowledge from both glycomics and glycoproteomics domains. For instance, in glycomics, a GlyTouCan ID has been assigned to each glycan structure as a unique identifier through the development of the glycan structure repository GlyTouCan. This has enabled glycomics researchers to precisely specify glycan structure information using the corresponding GlyTouCan IDs in their publications. Furthermore, each glycan structure entry in GlyTouCan can be associated with a publication related to that structure, making it possible to collect publications that contain references to the glycan structure with a particular GlyTouCan ID. In addition, glycoscience portal sites, such as GlyCosmos, integrate metadata such as the species in which the glycan structure was discovered, the name of the motif it contains, and various external resources describing the same structure, for each glycan structure registered in GlyTouCan. These results are available under the GlyCosmos Glycans (https://glycosmos.org/glycans) data resource. Recently, the collaboration between GlyCosmos and PubChem has also been enhanced (41) largely due to these GlyTouCan identifiers.

Like GlyTouCan, GlyComb is a data repository that allows unique identifiers to be assigned to each glycoconjugate entry, which we expect will enable the integration of knowledge linking glycomics with proteomics and other omics data in the future. We have shown how this was possible by investigating the entries registered from MS-Viewer and PRIDE. As a result, we found actually overlapping entries from both repositories, which was made possible by the GlyComb IDs assigned to them. We investigated these GlyComb IDs further to see if they happened to be submitted by the same user to both repositories, and we confirmed that while none of the duplicate entries originated from the same submitters, the exact same glycopeptides were reported. Moreover, we can compare the UniProt IDs for different entries. For example, the same UniProt ID is provided for the GlyComb entry numbers GC0170AE and GC017098 shown in Table 1, where we can see that they have the same peptide sequence "QQLQEQSAPPSKPDGQLQFR" and the same glycan modification on the serine residue at position 11. However, GC0170AE has an additional glycan modification on the serine residue at position 7. A similar relationship holds for the GC0170B3 and GC0173AC pair, which were found in one project, PXD035445 in PRIDE, and in two projects, PPV000002, and PPV000046 in MS-Viewer. These entries are all mucin *O*-glycan modifications, which are difficult to analyze. By integrating such glycoproteomics data with accession numbers, the whole picture can be examined, providing insight into the function of mucin *O*-glycans. As this work advances, we believe that the integration of knowledge from multiple omics fields (glycomics, proteomics, and glycoproteomics) will contribute to lowering the hurdle for multi-omics research in glycoscience.

What we consider to be the key to greatly improving the potential usefulness of GlyComb is to maximize the use of Semantic Web technologies. In GlyComb, each published glycoconjugate entry assigned a GlyComb ID is sequentially converted into a format called Resource Description Framework (RDF) (42) and stored in a database for Semantic Web technologies called a triplestore (43). This allows researchers to retrieve a variety of information scattered across multiple different databases at once, using a query language called SPARQL (44). For example, major biological pathway databases such as Reactome (45) and WikiPathways (46) have already published their pathway data in RDF. The pathway information in these databases could be combined with the glycoconjugate information stored in GlyComb using the SPARQL query language to find the corresponding GlyComb ID for each glycoconjugate entry in a pathway, enabling researchers to find out from GlyComb which species and which chemical pathways a particular glycoconjugate entry of interest appears in. Furthermore, it is expected to make it easier to obtain a comprehensive list of glycoconjugate entries related to a specific disease.

Moreover, subsumption relationships (47) are defined between multiple glycan structures in glycomics, and SPARQL can be used to retrieve more specific glycan structures from glycan structures with ambiguous linkage and monosaccharide information. Owing to this, the glycan composition information contained in each glycoconjugate entry in GlyComb will provide insight into what glycan structures are actually attached to the glycoconjugate molecule. In addition to glycan structures, it may be possible to define such relationships between glycopeptide entries in GlyComb based on the peptide sequence and modified glycan structures of each entry. In LC-MS/MS experiments, some of the peptide sequences resulting from protease treatment may be a complete subpart of other peptide sequences. In other cases, the peptide sequence may be exactly the same. However, only the modified glycan structures may be different. By defining the relationships among these corresponding entries, we may be able to discover new knowledge that has not been revealed before when integrating with various information such as pathway data using SPARQL. For example, in the overlapping UniProt IDs found in the common GlyComb entries in Table 1, we were able to find peptide sequences where one subsumed the other. Using Semantic Web technologies, such relationships can be defined, to integrate such knowledge into a whole. Consequently, it is expected that the use of SPARQL will enable us to gain insight into glycoconjugates by integrating multiple omics data, and conversely, it will enable access to glycoconjugate information from various omics fields, which will lead to a greater understanding of biological processes in general.

**Figure 7:**
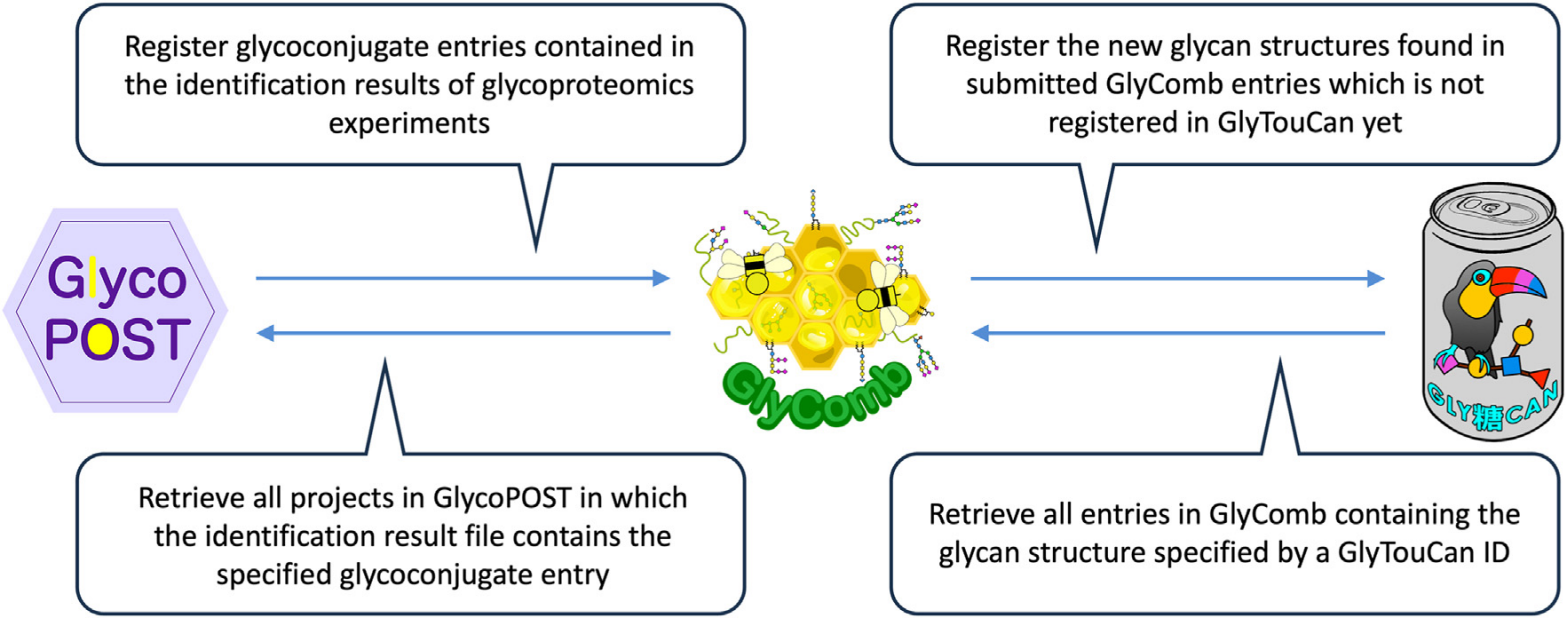
**Conceptual diagram showing an example of potential collaboration between three data repositories: GlyComb, GlycoPOST, and GlyTouCan.** Implementing a partnership system in GlyComb would allow for close collaboration among multiple data repositories, as shown in this figure. This allows users to easily jump from an entry in one data repository to the entries related to that entry in another data repository.

In the future, we plan to strengthen the collaboration between GlyComb and existing data repositories such as GlyTouCan, GlycoPOST, and UniCarb-DR in the glycosciences, and PRIDE, PubChem, LIPID MAPS (48), etc. in other omics fields. In order to extract the maximum amount of information from the results obtained from various studies, we plan to implement a partnership system into GlyComb. This system would allow, for example, data submitted to GlycoPOST containing the identification results of glycoconjugate data in an open file format, to be processed such that the relevant glycoconjugate results could be extracted and automatically registered in GlyComb. This would allow users to check the list of GlycoPOST entries in which a particular glycoconjugate molecule appears in the identification results via GlyComb IDs, or to check the list of GlyComb entries containing a particular glycan structure via GlyTouCan IDs. Figure 7 is a conceptual diagram showing an example of this partnership system between these three data repositories. We expect that GlyComb has the potential to unravel the hidden functions of glycoconjugate molecules by serving as a hub for integrating data from various omics fields, including glycomics, proteomics, and glycoproteomics.

## Experimental procedures

### Fundamental technologies used to build the GlyComb system

The GlyComb server-side system consists of several components. We developed most of the server-side logic, including the application programming interface (API), using the Java 17 programming language. As a relational database management system to directly store each entry submitted by users, we adopted the MariaDB Community Server. We also employed OpenLink Virtuoso Open Source Edition 7 for our implementation of a triplestore that stores the RDF-ized information converted from the relational database. For the RDF data insertion into this triplestore, we utilize SPARQList (https://github.com/dbcls/sparqlist). All these server-side components are running on our on-premise servers, which are built on containerized virtualization technology based on Docker (49).

To develop the front-end application, including the user interface that runs in web browsers, we used TypeScript for the majority of the development. We also used Node.js with a number of JavaScript libraries, including Webpack, to generate a JavaScript bundle file that web browsers can interpret. For the development of the conversion software to extract GlyComb input entries from the summary spreadsheet file generated from PMI-Byonic, we adopted the Scala programming language and used the Scala.js compiler to generate a JavaScript bundle file that runs in web browsers. Then, we embedded the bundle file in the front-end application so that it is available from the user interface.

### RDFization of data registered in GlyComb

**Figure 8:**
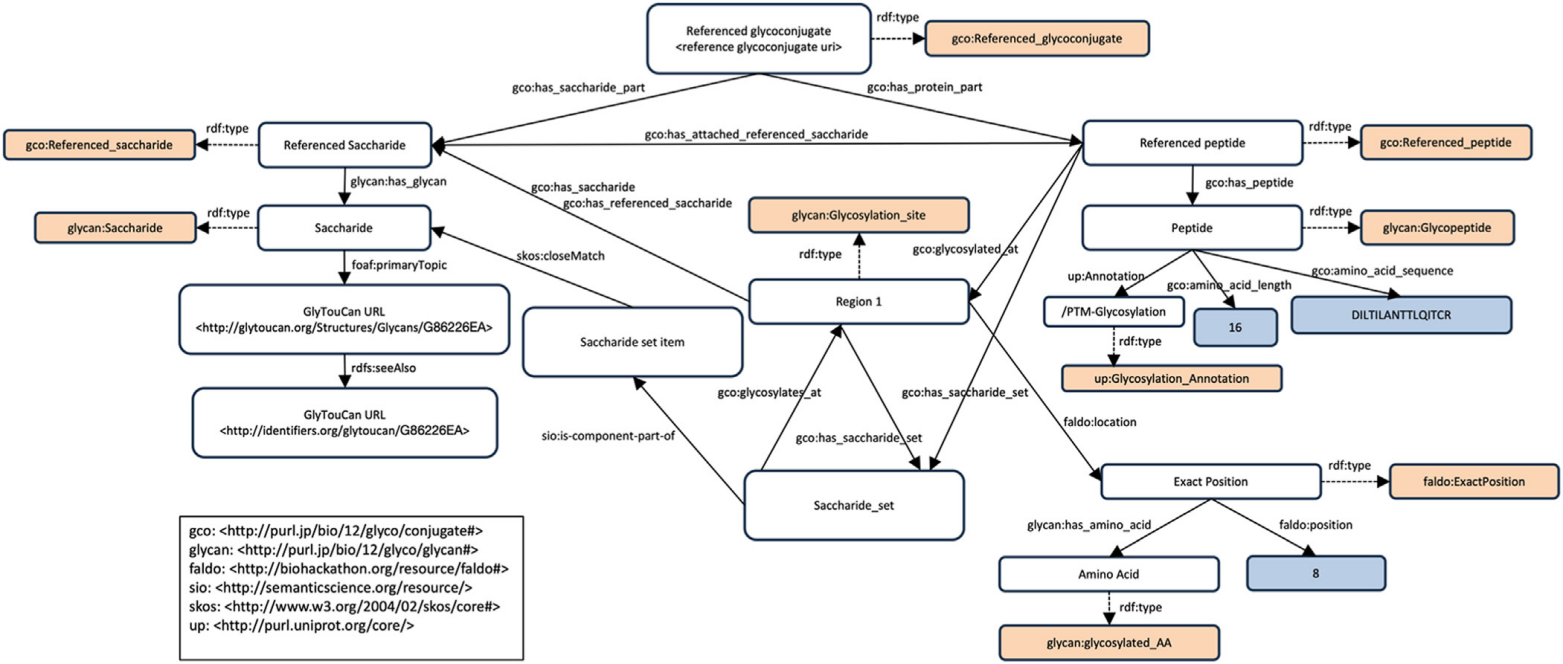
**RDF schema of GlyComb representing a glycopeptide entry with the glycan structure “HexNAc (4)Hex (7)NeuAc (1)” modifying the asparagine residue at residue number 8 of the peptide sequence “DILTILANTTLQITCR”.** This RDF data is part of a glycopeptide entry with GlyComb ID GC0017EF, which was derived from mouse vascular endothelial growth factor receptor 2 (VEGFR2) (UniProt ID: P35918). In this directed graph, each node represents a subject or an object, and a predicate is represented between the subject and the object as an edge in the subject-to-object direction. All glycan structure information entered by researchers is normalized to the corresponding GlyTouCan IDs and converted to RDF data through batch processing on the server. *Orange boxes* represent classes in RDF, and blue boxes represent literal values in RDF.

To convert the glycopeptide and glycoprotein entries submitted by researchers into RDF data that can be stored in a triplestore, we used the glycoconjugate ontology (GlycoCoO) (50), an ontology for standardizing glycoconjugate data as RDF data. This ontology is an extension of GlycoRDF (51) for standardizing glycomics information. Currently, GlyComb only stores glycopeptide and glycoprotein entries, whereas GlycoCoO is an ontology that can represent various glycoconjugate data including glycolipids. Therefore, we converted each entry of GlyComb into RDF data by adopting only a part of the definition of this GlycoCoO ontology. Figure 8 shows the RDF schema for representing a peptide sequence with only one glycosylated peptide residue as RDF data using GlycoCoO. In RDF, all data is encoded into a triple of subject, predicate, and object, which form a directed graph with the subject and object as nodes and the predicate as edges.

### Extraction of glycopeptide and glycoprotein information retrieved from PRIDE and MS-Viewer data repositories

We first fetched files from the FTP server of the PRIDE Archive using in-house Ruby scripts in order to retrieve glycopeptide and glycoprotein identification results from the 24,145 public PRIDE Archive submissions. Next, to collect the Excel files generated by PMI-Byonic from the retrieved files, we used a script written in the Rust programming language and the calamine package to scan all of the file contents. Finally, to extract the glycopeptide and glycoprotein information from the collected Excel files, we used our own conversion software as described in the previous section.

Subsequently, to retrieve the glycopeptide and glycoprotein identification results from the MS-Viewer repository in the Protein Prospector database, we created an in-house JavaScript program using the Puppeteer package and saved each identification result as a TSV file. We then created in-house Scala scripts to extract glycopeptide and glycoprotein information from these files. Finally, we also used in-house Python scripts to search and extract overlapping entries registered in GlyComb from both data repositories.

## Data availability

The manuscript contains all the data described within the text.

## Conflicts of interest

The authors declare that they have no conflicts of interest with the contents of this article.
